# Complete genome sequences of six coliphages isolated from local water systems in the Philippines

**DOI:** 10.1128/mra.00859-24

**Published:** 2024-11-29

**Authors:** Emilia Andrea V. Sabban, Adonis N. Eclipse, Stephen Kyle C. Arcan, Dexter Bryan L. Esliza, Paul Jeremy C. Lanete, Lindley C. Susi, Gelito Joseph M. Sikat

**Affiliations:** 1 Department of Science and Technology, Industrial Technology Development Institute, Taguig City, Philippines; 2 Department of Science and Technology, Science Education Institute, Taguig City, Philippines; DOE Joint Genome Institute, Berkeley, California, USA

**Keywords:** coliphages, whole-genome sequencing, Philippines, bacteriophages, *Felixounavirus*, Escherichia phages

## Abstract

Complete genome sequences of six coliphages (genomic size: 87–347 Kbp; GC content: 35.10%–45.27%) belonging to the *Felixounavirus*, *Tequintavirus*, and *Asteriusvirus* genera were isolated from Philippine wastewater facilities and drainage systems. These phages offer potential for development as diagnostic and therapeutic tools for food and water safety.

## ANNOUNCEMENT

To address pressing challenges in water and food safety, rapid and accurate on-site detection of bacterial pathogens is crucial. While various strategies are being explored, locally isolated bacteriophages offer a promising alternative to conventional methods of pathogen detection. Phage-based biosensors, known for their specificity, sensitivity, rapidity, cost-effectiveness, and portability, are particularly valuable in regions like the Philippines where immediate testing of bacterial contamination is imperative (1). Hence, developing innovative biosensors for enhanced public health is of great interest.

Wastewater collected from local drainage systems in Manila (July 2022) and sewage facilities in Zambales (January 2023) were enriched using *Escherichia coli* O157:H7 ATCC 43888. *E. coli* were grown in Luria Bertani broth (24 h, 37°C) prior to infection with isolated plaques at 150 rpm overnight. Phages were purified through three rounds of serial plaque isolations and were selected based on clear plaque morphology.

Genomic DNA was extracted from purified lysates using Promega viral total nucleic acid extraction protocol, followed by library preparation using Illumina DNA Prep and TruSeq DNA Nano (manufacturer’s protocols) (2). Subsequently, paired-end sequencing was done using the Illumina MiSeq platform with a 150 bp read length.

Sequence data analysis using software and tools was executed using default parameters unless otherwise specified. Raw sequence data underwent quality control using FastQC v0.12.1 (3). Adapter and PhiX sequences were removed using BBDuk (4) before *de novo* assembly with SPAdes v3.15.5 (5). The resulting assembly was visualized using Bandage (6) and assessed for completeness using CheckV v1.0.1 (7), confirming complete genomes, and QUAST v5.2.0 (8) for overall assembly metrics. Read mapping and error correction were handled with samtools v1.19.2 and Pilon v1.24 (9, 10).

**Fig 1:**
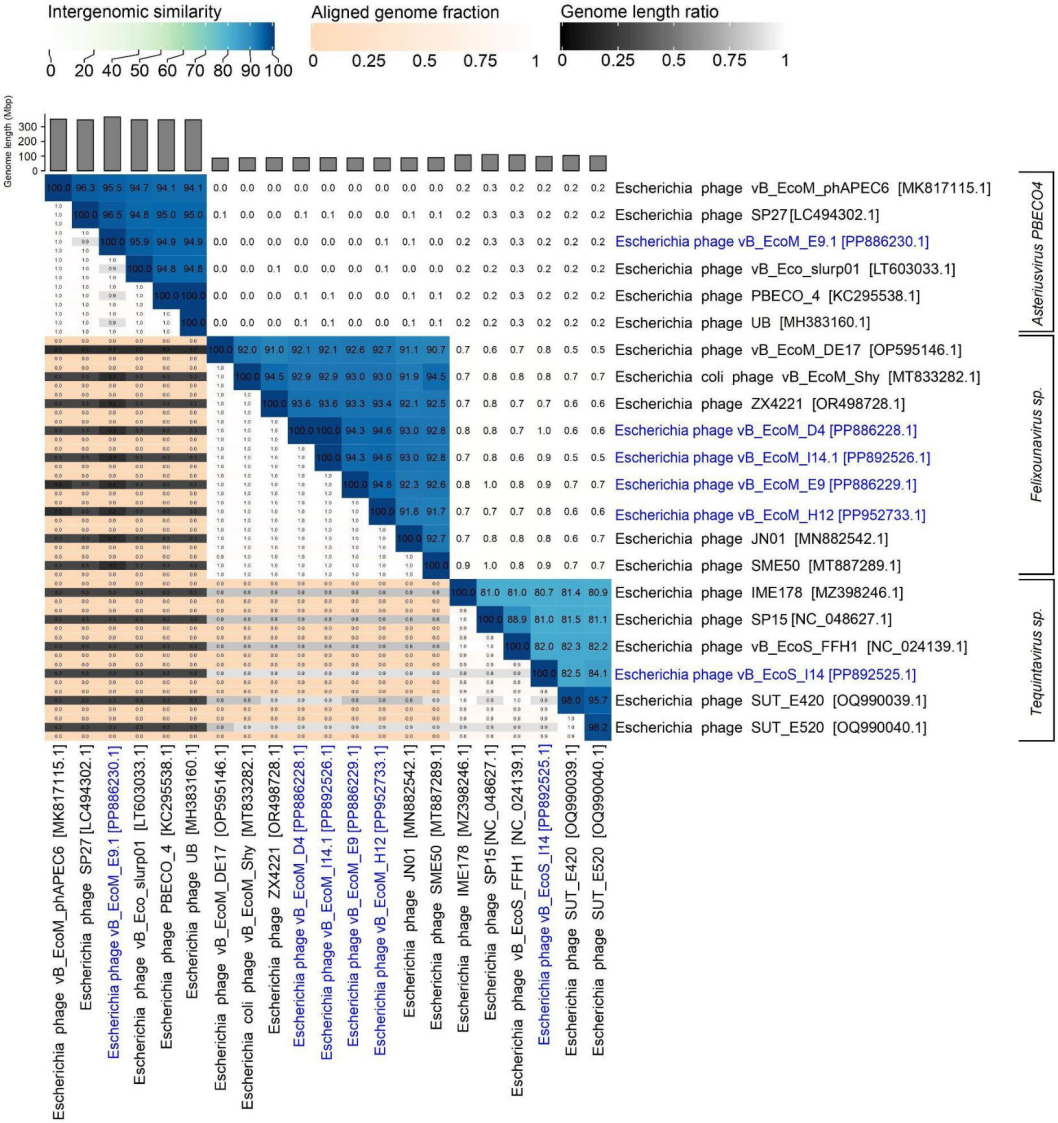
VIRIDIC-based intergenomic similarity analysis of six reported Escherichia phages. The heatmap illustrates the intergenomic relationships among the six assembled Escherichia phages (blue) and their closest reference genomes. Intergenomic similarity scores (upper half, darker shades indicate higher similarity), aligned genome fraction (orange to white, lighter shades indicate higher coverage), and genome length ratio (black to white, lighter shades indicate higher ratio) are presented. Genome lengths are represented by the bars above each column.

Preliminary identification was conducted using BLASTn against the NCBI nucleotide collection (nr/nt), with similarity scores calculated using the VIRIDIC algorithm (11, 12). To classify phages at the species level, a 95% intergenomic similarity threshold, as defined by the International Committee on Taxonomy of Viruses, was applied. Five of the six analyzed phages were categorized only up to the genus level: *Felixounavirus* (vB_EcoM_D4, E9, I14.1, and H12) and *Tequintavirus* (vB_EcoS_I14). Escherichia phage vB_EcoM_E9.1 was identified as *Asteriusvirus* PBECO4, exhibiting a 96.50% intergenomic similarity to its closest relative (Fig. 1). Notably, Escherichia phages vB_EcoM_D4 and I14.1 were determined to be identical at the species level, sharing 100% intergenomic similarity. Genomic characterization revealed size ranges of 87,697 to 367,851 base pairs, varying GC content from 35.10% to 45.27%, and coverage depths spanning 9.38 to 93.28× (Table 1). Phages E9 and E9.1; I14 and I14.1; were assembled from the same libraries, respectively.

**TABLE 1:** Summary of molecular features of six reported bacteriophage genomes

	Escherichia phage vB_EcoM_D4	Escherichia phage vB_EcoM_E9	Escherichia phage vB_EcoM_E9.1	Escherichia phage vB_EcoS_I14	Escherichia phage vB_EcoM_I14.1	Escherichia phage vB_EcoM_H12
No. of raw reads	1,519,100	1,537,068	1,537,068	1,449,058	1,449,058	3,663,770
No. of clean reads	674,152 (44.4%)	403,272 (26.23%)	403,272 (26.23%)	233,746 (16.13%)	233,746 (16.13%)	2,615,270 (71.39%)
Genome size (bp)	89,439	87,697	367,851	97,251	89,359	87,639
GC content (%)	35.94%	35.10%	35.10%	38.88%	38.88%	45.27%
Coverage	93.278156×	49.463293×	11.708068×	68.328727×	9.381270×	75.915163×
No. of CDS	149	149	731	169	150	146
No. of genes with predicted function	48 (32.21%)	46 (30.87%)	159 (21.75%)	69	48 (40.83%)	47 (32.19%)
No. of tRNAs	24	24	6	25	24	24
Lifestyle prediction	Lytic	Lytic	Lytic	Lytic	Lytic	Lytic
Taxonomic identity	*Felixounavirus* sp.	*Felixounavirus* sp.	*Asteriusvirus* PBECO4	*Tequintavirus* sp.	*Felixounavirus* sp.	*Felixounavirus* sp.
Temperate marker genes	–^*a*^	–	–	–	–	–
Presence of antibiotic resistance genes	–	–	–	–	–	–
Presence of virulence genes	–	–	–	–	–	–
BioProject accession no.	PRJNA1123411	PRJNA1123414	PRJNA1123416	PRJNA1123417	PRJNA1123418	PRJNA1126417
SRA accession no.	SRR30045796	SRR30123770	SRR30123770	SRR30124319	SRR30124319	SRR30123854
GenBank accession no.	PP886228	PP886229	PP886230	PP892525	PP892526	PP952733

^*a*^ '-' signifies the absence of the genes in the respective genomes.

Results from PhageTerm v1.0.11 for genome architecture analysis and determination of termini indicated that the phage genomes possessed direct terminal repeats and a linear structure (13). Functional annotation of the phage genomes was carried out using Pharokka v1.7.1. (14). PhageLeads was used to check for therapeutic suitability—no virulence factors, temperate lifestyle markers, nor antibiotic resistance genes were detected within the six coliphage genomes (15). The lytic lifestyle of these phages was confirmed through analysis with PhaTYP (16).

## Data Availability

All assembled sequences and raw reads were compiled and deposited into NCBI SRA and GenBank with accession numbers as listed in Table 1.
